# Relationship between Sleep and Hedonic Appetite in Shift Workers

**DOI:** 10.3390/nu12092835

**Published:** 2020-09-16

**Authors:** Parisa Vidafar, Sean W. Cain, Ari Shechter

**Affiliations:** 1 Sleep and Circadian Research Laboratory, University of Michigan, Ann Arbor, MI 48109, USA; pvidafar@med.umich.edu; 2 School of Psychological Sciences and Turner Institute for Brain and Mental Health, Monash University, Melbourne, Victoria 3800, Australia; sean.cain@monash.edu; 3 Center for Behavioral Cardiovascular Health, Columbia University Irving Medical Center, New York, NY 10032, USA; 4 Sleep Center of Excellence, Columbia University Irving Medical Center, New York, NY 10032, USA

**Keywords:** shift work, sleep, food intake, appetite, cravings, obesity, diet

## Abstract

Short and/or poor sleep are established behavioral factors which can contribute to excess food intake, and emerging evidence suggests that disturbed circadian rhythms may also impact food intake regulation. Together, disturbed sleep and circadian rhythms may help explain the excess risk for obesity seen in shift workers. To date, however, the details of how shift work may impact food intake regulation are still not fully defined. Here we examined the relationship between sleep characteristics and hedonic control of appetite in shift workers. A total of 63 shift workers (mean (M) age: 36.7 years, standard deviation (SD): 12.0; 59% women) completed an online survey comprising self-reported measures of body weight regulation, sleep (Pittsburgh Sleep Quality Index, Sleep Hygiene Index), and hedonic control of appetite (Food Craving Inventory, Power of Food Scale). Seventy-one percent reported some weight change since starting shift work, and 84% of those reported weight gain (M = +11.3 kg, SD = 9.1). Worse sleep quality and shorter sleep duration were associated with more food cravings, and worse sleep quality and hygiene were associated with higher appetitive drive to consume palatable food (greater hedonic drive). This preliminary study suggests hedonic pathways are potentially contributing to weight gain in shift workers with disturbed sleep.

## 1. Introduction

Sleep disturbance is recognized as a behavioral factor that can contribute to obesity [1]. Evidence for this link comes from both epidemiologic studies and mechanistic laboratory-based experiments, wherein short sleep and/or sleep restriction are associated with increased food intake [2]. The timing of the sleep–wake schedule is emerging as another factor which can impact body weight, with evidence that later sleep timing and sleep schedule irregularity can influence food intake regulation and adiposity [3,4,5].

The effects of disturbances to both sleep and circadian rhythms on obesity is particularly salient for shift workers who are at disproportionate risk for obesity and metabolic dysfunction. Indeed, in longitudinal analyses, shift work is associated with increases in body weight and adiposity [6,7]. This is likely due in part to the short sleep duration that is prevalent in shift workers [8], combined with the misalignment of circadian physiology with sleep–wake and feeding–fasting behaviors that occur as a necessary by-product of working atypical shift schedules. Limited availability of healthy food options [9,10] and fewer opportunities to engage in physical activity, [11] might also contribute to obesity in these individuals, and other shift work-related factors such as increased susceptibility to start smoking and higher rates of cigarette use [12,13] may also adversely impact health.

Experimental work demonstrated that simulating shift work conditions leads to a hormonal profile that would promote excess food intake [14,15]. In addition to this hormonal (homeostatic) control, an increasingly recognized mechanism linking impaired sleep and/or circadian rhythms with the risk of obesity is enhanced hedonic drive for food intake [2]. Neuroimaging studies have shown that brain regions involved with pleasure, reward, and motivation are disproportionately activated in response to food cues after disturbed sleep [16,17,18]. In terms of other assessments of the relationship of sleep with hedonic control of appetite, sleep restriction in adolescents resulted in impaired food-related inhibitory control and enhanced food reward [19]. We have also demonstrated that the relationship between poor sleep quality and body weight status is mediated by disinhibited eating behavior [20].

To our knowledge, no study has yet been done to examine the relationship between sleep and hedonic control of appetite in shift workers. We therefore administered an online questionnaire to examine how shift work affects body weight regulation and explored the relationship between sleep quality, duration, and hygiene and hedonic control of appetite in shift working individuals.

## 2. Materials and Methods

### 2.1. Participants

Participants were recruited through online advertisements on social media platforms, including Twitter, Facebook and Instagram. Interested individuals used the survey link or QR code on the study flyer to gain access to the online survey. Upon opening the online survey, participants were presented with the study’s explanatory statement and informed consent was provided by continuing the survey. All participants completed the survey anonymously, typically within 30 min or less. Participants were required to be 18 years or older, speak English and engage in shift work schedules. Procedures were approved by the Monash University Human Research Ethics Committee (project identification code: 20526; date of approval: 12/2/2019). A total of 63 shift workers were included in the current analyses.

### 2.2. Procedure

Sleep quality was measured using the Pittsburgh Sleep Quality Index (PSQI), a 19-item self-reported questionnaire based on sleep-related behaviors and experiences during the past month [21]. The PSQI measures seven sleep-related components: subjective sleep quality, sleep latency (time it takes to fall asleep), sleep duration (actual time spent sleeping), sleep efficiency (percentage of time spent sleeping while in bed), sleep disturbances, use of sleep medications and daytime dysfunction. Global scores range from 0–21, with >5 indicating clinically significant poor sleep quality [21].

Sleep hygiene (a collective term for behaviors and habits that promote good sleep quality) was measured using the Sleep Hygiene Index (SHI) [22], a 13-item, self-reported questionnaire assessing behavioral (e.g., I use alcohol, tobacco, or caffeine within 4 h of going to bed or after going to bed) and environmental (e.g., I sleep on an uncomfortable bed) factors that can lead to poor sleep quality [23]. Total scores are out of 52, with higher scores indicating poorer sleep hygiene. Sleep hygiene has been shown to have a strong association with sleep quality, and these behaviors appear to remain stable over time in the general population [23].

Shift worker food cravings were measured using the Food Craving Inventory (FCI), which is a 28-item self-reported questionnaire assessing craving for specific highly-palatable foods (four subscales include sweets, carbohydrates/starches, high fat foods and fast food fats) over the past month. We computed FCI scores for each participant by taking an average of all responses from the 28-items on the questionnaire. Higher scores indicate greater food cravings [24].

The 15-item Power of Food Scale (PFS) was used to measure hedonic control of food intake [25]. The PFS measures an individual’s appetite towards palatable foods by asking them to rate the extent to which they relate to each statement on a 5-point Likert scale from 1 (don’t agree at all) to 5 (strongly agree). The 15-item statements fall into three domains of proximity to the food source: (1) food that is readily available in the environment but not physically present (e.g., I find myself thinking about food even when I’m not physically hungry), (2) food that is physically present but has not been tasted (e.g., if I see or smell a food I like, I get a powerful urge to have some) and (3) food that has been tasted but not consumed (e.g., when I eat delicious food, I focus a lot on how good it tastes) [26]. Higher scores indicate a greater appetitive drive to consume highly palatable foods [25].

### 2.3. Data Analysis

Data were collected using Qualtrics software, version (08/2019) (Qualtrics, Provo, UT, USA). Pearson’s correlations followed by simple linear regressions analyses were performed using GraphPad Prism version 8.4.2 for MacOS (GraphPad Software, San Diego, CA, USA).

## 3. Results

Participants were 18–68 years old (mean (M) = 36.7, standard deviation (SD) = 12.0), comprising 37 cisgender women and 26 cisgender men (*n* = 63 total). Participants engaged in various shift work schedules, including night shifts (17%), forward rotating shifts (defined as shifts that change from day to afternoon to night [27,28]; 24%), backward rotating shifts (defined as shifts that change from day to night to afternoon [27,28]; 10%), early morning shifts (10%), evening/swing shifts (6%), on-call (3%) and other (e.g., 24 h on/24 h off; 30%). Four main sectors of the workforce were identified: emergency responders (e.g., firefighters), health care workers (e.g., nurses), goods and services (e.g., bartenders), and transport (e.g., truck drivers; see Table 1 for demographics summary).

Self-reported body weight maintenance metrics are shown in Table 2. Overall, 83% of participants reported that shift work affects their body weight maintenance in general. Fifty percent or more of participants in each shift work type reported that shift work affects weight maintenance (i.e., a particular shift work schedule such as night shifts does not seem to be differentially affecting weight maintenance; Table 2). Seventy-one percent of participants reported some weight change since they began working shift schedules. Of these, 84% of the participants reported weight gain (M = +11.3 kg, SD = 9.1) and 16% reported weight loss (M = −11.0 kg, SD = 12.9). Sixty percent of participants attempted weight loss since starting shift work, and of those, 34%, or 21% of the total sample, were successful in their weight loss attempts.

Overall, the average PSQI global score was 8.54 (SD = 3.47) and 87% of participants reported poor sleep quality (PSQI > 5). Participants slept an average of 6.38 h (SD = 1.38), with sleep duration derived from item four on the PSQI. Sixty-two percent of participants reported sleeping less than 7 h per night. The average score on the SHI was 20.68 (SD = 7.21). On average, participants scored 2.04 (SD = 0.47) on the FCI, and an average score of 36.11 (SD = 15.13) on the PFS.

**Figure 1:**
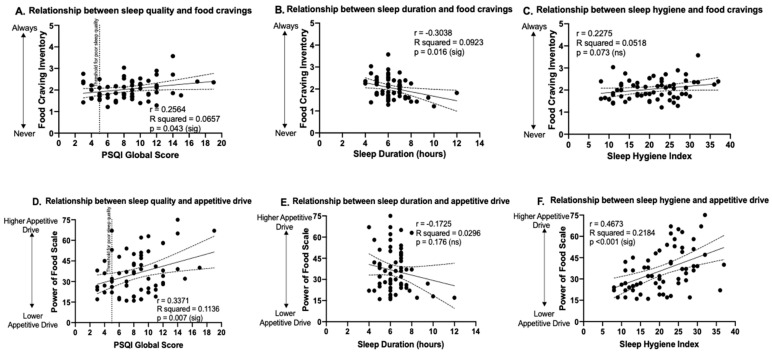
The relationship between sleep measures and food cravings and appetitive drive. Relationships of sleep measures to Food Craving Inventory scores are shown in the top panels (**A**–**C**) and relationships of sleep measures to Power of Food Scale scores are shown in the bottom panels (**D**–**F**). Vertical dotted line in (**A**) and (**D**) indicates threshold for clinically significant poor sleep quality (PSQI global score >5). Dashed lines above and below the fitted regression lines indicate the 95% confidence interval. Statistically significant *p*-values (*p* < 0.05) are denoted as (sig) and non-significant results (*p* ≥ 0.05) denoted as (ns). PSQI: Pittsburgh Sleep Quality Index.

Simple linear regression was used to predict hedonic appetite measures based on sleep measures. A statistically significant positive association was found between PSQI score and FCI score (F_1,61_ = 4.29, *p* = 0.04, *r* = 0.26; Figure 1A), indicating worse sleep quality was associated with higher cravings. A statistically significant negative association was found between sleep duration and FCI (F_1,61_ = 6.20, *p* = 0.02, *r* = −0.30; Figure 1B). The association between sleep hygiene and FCI was not statistically significant (F_1,61_ = 3.33, *p* = 0.07, *r* = 0.23; Figure 1C). Simple linear regressions carried out to assess whether sleep quality, sleep duration and sleep hygiene predicted appetitive drive showed that PSQI score had a significant positive association with PFS score (F_1,61_ = 7.819, *p* = 0.007, *r* = 0.34; Figure 1D), indicating worse sleep quality was associated with higher appetitive drive; sleep duration was not significantly associated with PFS scores (F_1,61_ = 1.87, *p* = 0.176, *r* = −0.17; Figure 1E); and sleep hygiene was significantly associated with PFS scores (F_1,61_ = 17.05, *p* = 0.0001, *r* = 0.47; Figure 1F), indicating worse sleep hygiene was associated with higher appetitive drive.

## 4. Discussion

The purpose of this study was to examine how shift work affects body weight regulation and to explore the relationship between sleep quality, duration, and hygiene, with hedonic control of appetite. We found that the majority of individuals reported shift work affecting their ability to maintain their body weight. In those who reported weight change since starting shift work, reports of weight gain far exceeded the reports of weight loss. Worse sleep quality and shorter sleep duration were associated with more food cravings, assessed via the FCI. We also found that worse sleep quality and hygiene were associated with higher appetitive drive to consume palatable food (greater hedonic drive), as measured with the PFS. We did not observe a relationship between sleep hygiene and food cravings or sleep duration and hedonic control of appetite.

Our finding that poor sleep quality is associated with more food cravings aligns with the findings from an earlier study conducted in healthy adults with a parental history of type 2 diabetes, which showed an association between poor sleep quality and increased levels of hunger and emotional and uncontrolled eating [29]. Consecutive nights of short sleep duration (5 h) have also been shown to increase food intake in excess of the energy an individual requires, another potential contributor to the weight gain observed in shift workers [30]. Our finding that short sleep duration is associated with more food cravings is also consistent with other cross-sectional studies [20,31].

The novel aspect of this study lies in its assessment of how sleep measures relate to the hedonic control of appetite in shift workers. We found that poor sleep quality and poor sleep hygiene were predictors of higher hedonic appetitive drive in our cross-sectional sample of shift workers. Previous cross-sectional work in healthy adolescents has shown that when individuals of normal weight are put on a restricted sleep schedule of 5 h per night, compared to 9 h per night, they exhibit less inhibitory control of food intake and find food to be more rewarding [19]. The authors suggest that this is likely attributable to the poor judgement of what constitutes healthy food items when acutely sleep deprived. It is well established that sleep deprivation impairs executive function, leading to poorer decision making in individuals experiencing acute or chronic sleep loss [32,33,34,35].

Most studies on the role of sleep in food intake regulation have focused on homeostatic (i.e., hormonal) systems, with relatively less exploring hedonic, or reward-driven, pathways [36]. This is important, since evidence from laboratory-based studies assessing ad libitum food intake and appetite/hunger-regulating hormones in the context of experimental sleep restriction, suggests that excess energy intake following short sleep is likely driven by hedonic as opposed to purely hormonal factors [37]. Thus, a strength of the current work was that we included measures of hedonic drive of appetite. Moreover, this is, to our knowledge, the first time hedonic drive of appetite has been assessed in shift workers.

An important limitation is that data were cross-sectional. Thus, we cannot determine a causal relationship between disturbed sleep in shift workers and body weight gain. Additionally, participants in our sample were more inclined to be overweight and obese. On average, women in our sample had overweight (body mass index 25–29.9 kg/m^2^), while on average the men had obesity (body mass index ≥ 30 kg/m^2^), thus limiting some generalizability. We also relied on self-report measures of sleep and of body weight. Future work should include behavioral measures of hedonic control of food intake in shift workers, for example via the “sipometer,” a device we have previously used to quantify food reward value and motivation to consume under sleep restriction conditions [38].

The current sample size was small, and although our sample included both males and females, this small sample size precludes sex-based comparisons. There is some evidence, including findings from experimental work, that females could be more susceptible than males to the adverse impact of shift work on metabolic outcomes [39]. A larger follow-up study powered to detect differences between male and female shift workers can help inform more personalized approaches to maintaining body weight and metabolic health in shift workers.

Overall, this is an early report that should be considered as hypothesis-generating and which suggests novel hedonic-focused pathways contributing to weight gain in shift workers with disrupted sleep. This preliminary study provides insight into hedonic factors potentially contributing to obesity risk in shift workers, who may have heightened responsivity to food intake as a result of disturbed sleep and/or potentially, circadian misalignment.

## Figures and Tables

**Table 1:** Participant demographics.

	**Sample Size (*n*)**	**Mean (Standard Deviation) or %**
Age (years)	63	36.7 (12)
Females	37	59%
Males	26	41%
Body mass index, Females		28.3 (8.2) kg/m^2^
Body mass index, Males		31.7 (11.5) kg/m^2^
**Work schedule type**		
Night shifts	11	17%
Forward rotating shifts	15	24%
Backward rotating shifts	6	10%
Early morning shifts	6	10%
Evening/swing shifts	4	6%
On-call	2	3%
Other (e.g., 24 h on, 24 h off)	19	30%
**Workforce sector**		
Emergency responders	21	33%
Health care workers	26	41%
Goods and services	6	10%
Transport	9	14%

**Table 2:** Self-reported body weight management metrics.

	YES	NO	N/A
**Shift Work Affects Weight Maintenance**	83% (*n* = 52)	17% (*n* = 11)	--
Night shifts (*n* = 11)	73% (*n* = 8)	27% (*n* = 3)	
Forward rotating shifts (*n* = 15)	73% (*n* = 11)	27% (*n* = 4)	
Backward rotating shifts (*n* = 6)	83% (*n* = 5)	17% (*n* = 1)	
Early morning shifts (*n* = 6)	67% (*n* = 4)	33% (*n* = 2)	
Evening/swing shifts (*n* = 4)	50% (*n* = 2)	50% (*n* = 2)	
On-call (*n* = 2)	100% (*n* = 2)	0% (*n* = 0)	
Other (e.g., 24 h on, 24 h off; *n* = 19)	68% (*n* = 13)	32% (*n* = 6)	
**Weight change since starting shift work**	71% (*n* = 45)	29% (*n* = 18)	--
Weight gain: 84%, *n* = 38, +11.3 (9.1) kg			
Weight loss: 16%, *n* = 7, −11.0 (12.9) kg			
Weight loss attempt since shift work	60% (*n* = 38)	8% (*n* = 5)	32% (*n* = 20)
Successful weight loss attempt	21% (*n* = 13)	41% (*n* = 26)	38% (*n* = 24)

Data are expressed as % (*n*) or mean (standard deviation); N/A: Not applicable.
